# Diagnostic and Prognostic Value of Serum Glial Fibrillary Acidic Protein in Acute Ischemic Stroke

**DOI:** 10.3390/jcm15051971

**Published:** 2026-03-04

**Authors:** Luisa Agnello, Anna Maria Ciaccio, Fabio Del Ben, Mario Daidone, Gaetano Pacinella, Anna Masucci, Martina Tamburello, Caterina Maria Gambino, Antonino Tuttolomondo, Marcello Ciaccio

**Affiliations:** 1 Department of Biomedicine, Neurosciences and Advanced Diagnostics, Institute of Clinical Biochemistry, Clinical Molecular Medicine, and Clinical Laboratory Medicine, University of Palermo, 90127 Palermo, Italy; luisa.agnello@unipa.it (L.A.); anna.masucci@unipa.it (A.M.); martina.tamburello@unipa.it (M.T.); caterinamaria.gambino@unipa.it (C.M.G.); 2 Department of Laboratory Medicine, University Hospital Paolo Giaccone, 90127 Palermo, Italy; 3 Department of Health Promotion, Mother and Child Care, Internal Medicine and Medical Specialities, University of Palermo, 90127 Palermo, Italy; annamaria.ciaccio@unipa.it (A.M.C.); mario.daidone@unipa.it (M.D.); gaetano.pacinella@unipa.it (G.P.); antonino.tuttolomondo@unipa.it (A.T.); 4 Immunopathology and Cancer Biomarkers, Centro di Riferimento Oncologico (CRO)-IRCCS, 33081 Aviano, Italy; fabio.delben@cro.it

**Keywords:** acute ischemic stroke, GFAP, biomarker, diagnosis, prognosis, NIHSS

## Abstract

**Background:** Acute ischemic stroke (AIS) remains a major cause of morbidity and mortality, with an unmet need for reliable blood-based biomarkers. Glial fibrillary acidic protein (GFAP), an astrocytic structural protein, is established in hemorrhagic stroke and traumatic brain injury, but its role in AIS remains incompletely defined. **Methods:** In this retrospective case-control study, we enrolled AIS patients and healthy controls. Serum GFAP was measured within 24 h using the Lumipulse G1200 automated assay. Stroke severity and outcome were assessed with the National Institutes of Health Stroke Scale (NIHSS) and functional outcome with the modified Rankin Scale (mRS). Associations with clinical measures were explored using Spearman correlation, and diagnostic accuracy was determined by ROC analysis. **Results:** GFAP levels were significantly higher in AIS patients than controls (median 132.9 vs. 30.0 pg/mL, *p* < 0.001). The ROC analysis yielded an AUC of 0.88 (95% CI 0.81–0.96). A cutoff of 71 pg/mL achieved 74% sensitivity and 92% specificity, while 150 pg/mL and 32 pg/mL optimized positive and negative predictive values (95% and 96%). GFAP was correlated with stroke severity (NIHSS, ρ = 0.37–0.40, *p* < 0.001) and disability (mRS, ρ = 0.48–0.49, *p* < 0.001). No significant differences appeared across TOAST subtypes. **Conclusions:** Serum GFAP is significantly elevated in AIS and demonstrates strong diagnostic and prognostic value. Integration of GFAP into clinical workflows may enhance early stroke detection and outcome prediction, supporting its role as a promising biomarker in AIS.

## 1. Introduction

Acute ischemic stroke (AIS) represents a major global health challenge, ranking as the third leading cause of death and the fourth leading cause of disability-adjusted life years worldwide [1]. In 2021, there were approximately 7.8 million incident cases of ischemic stroke globally, accounting for 65.3% of all stroke subtypes [1,2]. Despite declining age-standardized incidence rates in some regions, the absolute burden continues to rise due to population growth and aging, with projections indicating further increases through 2030 [3,4]. This growing burden underscores the urgent need for improved diagnostic and prognostic tools to optimize patient management and outcomes.

Current stroke diagnosis relies heavily on neuroimaging, which, while effective, is expensive, not universally available, and can be time-consuming in emergency settings where rapid treatment decisions are critical [5]. Blood-based biomarkers offer a complementary approach that could expedite diagnosis, aid in stroke subtype differentiation, predict clinical outcomes, and potentially guide therapeutic decisions [6,7]. However, despite extensive research, no individual biomarker or panel has achieved sufficient diagnostic performance for routine clinical use in the acute setting, particularly within the narrow therapeutic window for interventions such as thrombolysis [5,8].

In the last decade, Glial fibrillary acidic protein (GFAP) has emerged as a promising biomarker for acute brain injury. It is an intermediate filament protein expressed in astrocytes and released into the bloodstream following brain injury. While elevated GFAP levels are well documented in hemorrhagic stroke, traumatic brain injury, and neurodegenerative diseases, such as Alzheimer’s disease, increasing evidence suggests that GFAP may also play a role in the pathophysiology and clinical course of ischemic stroke [9,10,11,12]. Nevertheless, the diagnostic and prognostic value of GFAP in AIS remains incompletely defined, with conflicting results across different populations and methodologies. GFAP reflects astrocytic injury and blood–brain barrier disruption and may provide additional information to established clinical assessments or neuroimaging.

The present study aimed to explore the value of serum GFAP as both a diagnostic and prognostic biomarker in patients with AIS. Specifically, we sought to (1) determine the diagnostic accuracy of GFAP for distinguishing AIS patients from healthy controls and identify clinically relevant cutoff thresholds; (2) assess whether GFAP levels differ among TOAST etiological subtypes; and (3) examine the correlation between GFAP concentrations and both stroke severity (NIHSS) and functional disability (mRS) at multiple time points during hospitalization. By addressing these objectives using a well-characterized cohort and a validated automated GFAP assay, we aimed to provide evidence supporting the integration of GFAP into multimodal approaches for stroke evaluation and risk stratification.

## 2. Materials and Methods

### 2.1. Study Population

In this case-control retrospective observational study, we enrolled 59 consecutive patients with AIS admitted from June 2021 to January 2024 to the Internal Medicine Ward with Stroke Care at the University Hospital “Paolo Giaccone” in Palermo. Controls were consecutive healthy blood donors from the Unit of Transfusion Medicine of the Policlinico Paolo Giaccone of Palermo.

Ischemic stroke was defined as an episode of neurological dysfunction caused by focal cerebral infarction lasting more than 24 h. For each patient, we evaluated their medical history, 12-lead ECG, 24 h electrocardiography monitoring, trans-thoracic echocardiography, carotid ultrasound, brain CT, or MRI results at admission. Inclusion criteria for acute ischemic stroke patients were (1) age ≥ 18 years; (2) admission with symptoms suggestive of acute cerebral ischemia; (3) diagnosis of acute ischemic stroke confirmed by clinical evaluation and neuroimaging; (4) availability of blood sampling for GFAP measurement obtained during the acute phase within a predefined time window from symptom onset; and (5) written informed consent obtained from the patient or a legally authorized representative. Exclusion criteria were rheumatologic disorders, chronic inflammatory disease, acute systemic infections, recent venous thrombosis, recent acute myocardial infarction (AMI) (within 3 months), and recent cerebrovascular event (TIA or stroke within 6 months). Stroke etiology was categorized according to the Trial of ORG 10172 in Acute Stroke Treatment (TOAST) classification, which includes large-artery atherosclerosis (LAAS), cardioembolism (CEI), small-vessel occlusion (LAC), stroke of other determined etiology (ODE), and stroke of undetermined/multiple etiologies (UDE) [13]. Stroke severity was assessed using the NIHSS on admission (day 0), on day 6, and at hospital discharge. Functional outcome was measured using the mRS both on admission and at discharge. All patients were managed with antiplatelet therapy.

Healthy controls were adult blood donors with no history of stroke or other acute or chronic neurological disease, no acute illness at the time of donation, and no known inflammatory or malignant conditions.

All participants signed informed consent. The study was conducted in accordance with the Declaration of Helsinki and approved by the Institutional Review Board (ethics committee) of Policlinico P. Giaccone Palermo (V. n.7/2020).

### 2.2. Biochemical Analysis

For patients, venous blood samples were collected in dry tubes within 24 h after stroke onset. Samples were centrifuged at 3000× *g* for 10 min within 1 h of collection, and the separated serum was aliquoted and stored at −80 °C until analysis.

Serum GFAP levels were measured by the Lumipulse G GFAP assay on the fully automated platform Lumipulse G1200 (FUJIREBIO Inc., Tokyo, Japan). This automated assay has a limit of detection of 1.8 pg/mL, a limit of quantification at 10% CV, and a precision of less than 5% coefficient of variation (CV) [14].

As a reference standard, the assay uses full-length recombinant human GFAP protein (LS Bio, Shirley, MA, USA). The detection range is 4.0–5000.0 pg/mL.

### 2.3. Statistical Analysis

All statistical analyses were performed using R software (version 4.5.1; R Foundation for Statistical Computing, Vienna, Austria). To minimize the impact of demographic confounders, age- and sex-matched cohorts were generated using the MatchIt package with exact matching. Normality of continuous variables was assessed with the Shapiro–Wilk test and visual inspection of distribution plots. Since GFAP values and clinical scores showed non-normal distributions, data were expressed as medians with interquartile ranges (IQR), and non-parametric tests were applied.

Comparisons of continuous variables between two groups were conducted using the Mann–Whitney U test, while comparisons across more than two groups were assessed with the Kruskal–Wallis test followed by post hoc pairwise testing when appropriate.

Correlations between serum GFAP levels and clinical measures (NIHSS and mRS) were evaluated using Spearman’s rank correlation coefficient (ρ) with 95% confidence intervals. *p*-values for multiple correlations were adjusted with Benjamin–Hochberg correction.

The diagnostic performance of GFAP was assessed by receiver operating characteristic (ROC) curve analysis, with area under the curve (AUC) estimates and 95% confidence intervals using 2000 bootstrap replicates.

Data visualization and statistical testing were performed using the ggstatsplot 0.13.1 and pROC 1.19.0.1 packages. A two-tailed *p* value < 0.05 was considered statistically significant.

## 3. Results

### 3.1. Study Population

In this study, we enrolled a total of 397 participants, including 59 patients with AIS and 338 healthy controls. There was no significant difference in sex distribution between the cases and controls, whereas the patients were significantly older than the controls. To minimize confounding by demographic variables, exact age and sex matching was performed, yielding a comparison cohort of 131 individuals (38 AIS patients and 93 controls).

The AIS patients had a median age of 74 years, and 61% were male. The most frequent TOAST subtype was large-artery atherosclerosis (n = 25), followed by cardioembolism (n = 17), small-vessel occlusion (n = 8), undetermined etiology (n = 7), and other determined etiology (n = 2).

During hospitalization, the patients exhibited significant neurological improvement. The median NIHSS score declined from 5.5 on admission to 3.5 at day 6, and further to 2.5 at discharge (*p* < 0.001 across time points). Functional status, assessed by the mRS, similarly improved from a median of three at baseline to two at discharge (*p* < 0.001).

### 3.2. GFAP as a Diagnostic Biomarker in Acute Ischemic Stroke

**Figure 1:**
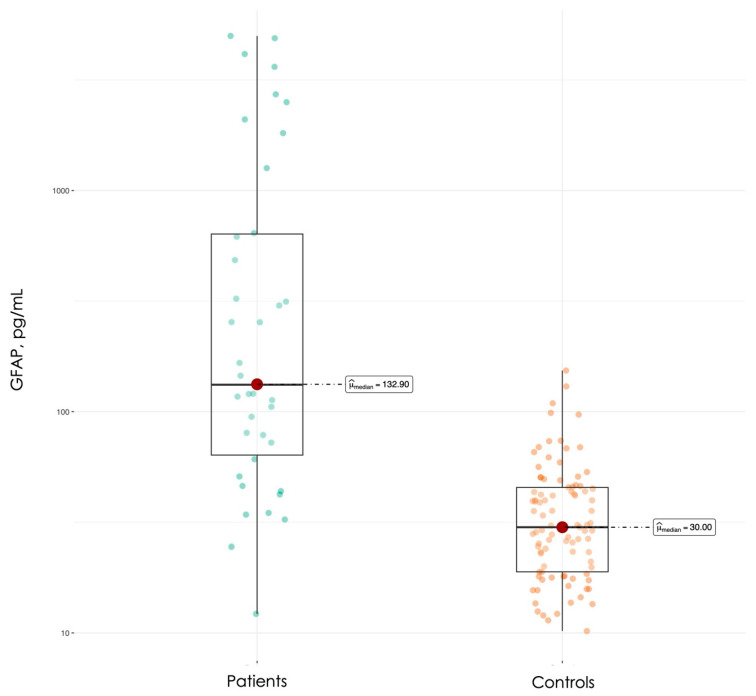
Serum GFAP levels in patients and controls.

**Figure 2:**
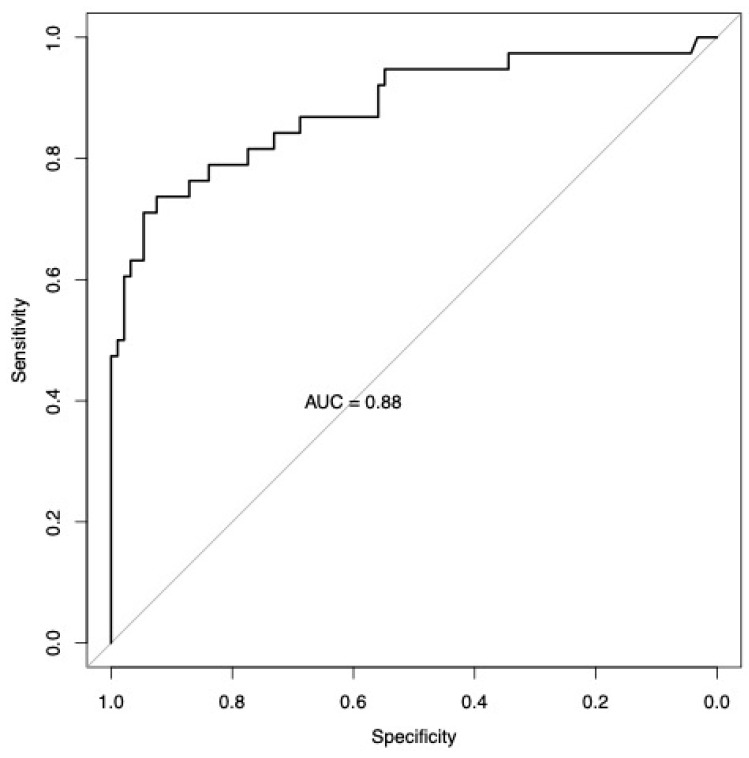
ROC curve analysis of serum GFAP for detecting acute ischemic stroke.

Serum GFAP levels were significantly higher in AIS patients compared with healthy controls (median 132.9 vs. 30.0 pg/mL, *p* < 0.001) (Figure 1). ROC curve analysis showed an AUC of 0.88 (95% CI 0.81–0.96) (Figure 2). The Youden’s index cutoff was 71 pg/mL, yielding a sensitivity of 74% CI 58–87, specificity of 92% CI 86–97, positive predictive value (PPV) of 80% CI 68–91, and negative predictive value (NPV) of 90% CI 84–95. For a decision support cutoff prioritizing confidence in disease detection, a threshold of 150 pg/mL was identified, achieving a PPV of 95% CI 83–100 and an NPV of 82% CI 78–87, with a sensitivity of 47% CI 32–63 and a specificity of 99% CI 97–100. For ruling out disease with high confidence, a threshold of 32 was identified, providing an NPV of 96% CI 90–100.

### 3.3. GFAP as a Prognostic Biomarker in Ischemic Stroke

**Figure 3:**
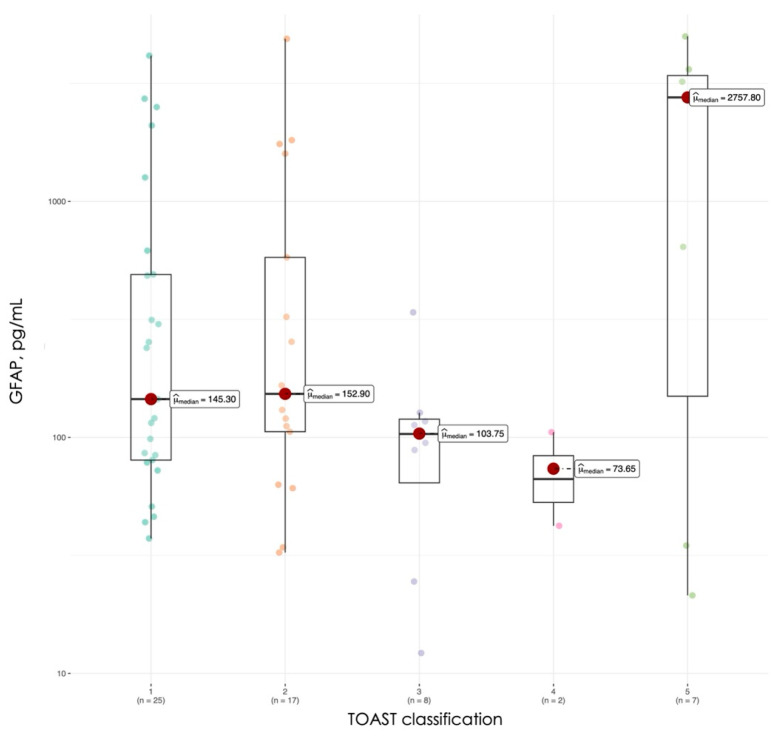
GFAP levels in patients according to TOAST classification.

GFAP levels did not significantly vary among TOAST subtypes, although a nonsignificant trend in subtype 5 showing the highest levels is visible, especially vs. subtypes 3 and 4. The low number of patients might have affected the significance (Figure 3).

**Figure 4:**
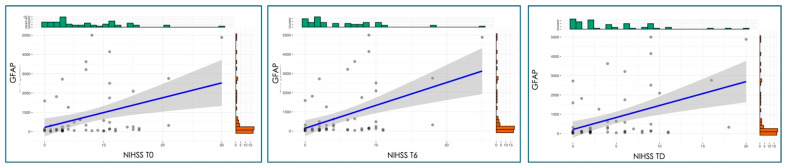
Correlation analysis between GFAP and NIHSS at different time points. T0, at admission; T6, after six days of hospitalization; and TD, at discharge.

**Figure 5:**
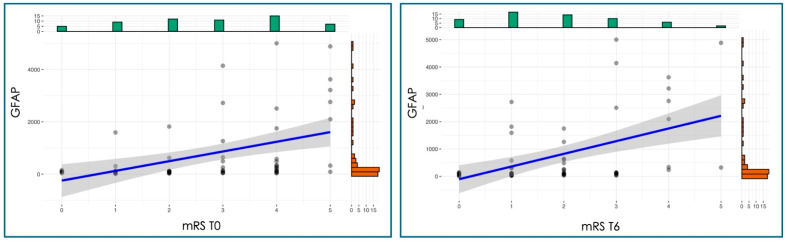
Correlation analysis between GFAP and mRS at different time points. T0, at admission; T6, after six days of hospitalization.

GFAP levels showed a significant moderate-to-strong positive relationship with stroke severity at all time points (*p* < 0.01), as measured by the NIHSS (Figure 4), and with functional disability assessed by the mRS at all time points (*p* < 0.001) (Figure 5). The Spearman coefficient was 0.37–0.40 in the case of NIHSS and 0.48–0.49 in the case of MRS.

### 3.4. Univariate and Multivariate Regression Analysis

Referring to MRS at discharge as the outcome, a univariate regression for each of the available confounders was performed to rule out non-significant associations and reduce the number of variables to be included in the multivariate regression (Table 1). Infarct volume was not available, but clinical severity scores at admission are a good proxy (functional limitation is proportional to infarct volume). Only GFAP, hypercholesterolemia, NIHSS, and MRS at admission showed significant association with MRS at discharge. A similar pattern was found when evaluating NIHSS at discharge. When including all these variables in a multivariate regression, only MRS at admission showed an independent association (*p* < 0.001). In multivariate models in which the same clinical scale was used as both predictor and outcome (e.g., NIHSS at admission predicting NIHSS at discharge), and the other clinical scale was not used, GFAP showed a non-significant trend (*p* = 0.07 for NIHSS; *p* = 0.10 for mRS). However, when using different clinical metrics at admission with respect to discharge, GFAP emerged as an independent predictor of functional outcome (*p* = 0.02) in the model adjusting for NIHSS at admission to predict mRS at discharge. Conversely, no significant independent association was found when mRS at admission was used to predict NIHSS at discharge.

## 4. Discussion

In this retrospective observational study, we investigated the diagnostic and prognostic role of serum GFAP in patients with acute ischemic stroke. This study demonstrates that serum GFAP measured within 24 h of symptom onset exhibits both diagnostic and prognostic value in acute ischemic stroke. The findings confirm GFAP as a blood-based biomarker that can distinguish AIS patients from healthy controls with good diagnostic accuracy (AUC 0.88) and reveal significant correlations between GFAP levels and both stroke severity and functional disability throughout hospitalization. GFAP exhibited a non-significant trend when predicting outcomes within the same clinical domain, suggesting a marginal but insufficient (possibly due to the limited sample size) incremental value. However, when both clinical scales were included simultaneously in the multivariate model, the association of GFAP was entirely lost. This indicates that while GFAP carries relevant biological information, its signal becomes redundant when the combined clinical picture, encompassing both neurological deficit (NIHSS) and functional status (mRS), is fully accounted for.

Interestingly, the independent prognostic value of GFAP was maintained alongside NIHSS at admission when predicting mRS at discharge (*p* = 0.02). This suggests that the biomarker provides additional information on the biological substrate of injury that contributes to functional disability. In contrast, when using mRS at admission to predict NIHSS at discharge, the clinical score overshadowed all other variables, including GFAP. This finding likely reflects the fact that mRS at admission captures the patient’s pre-stroke functional status and global frailty, which have a dominant ceiling effect on their potential for neurological recovery, thereby masking the subtler prognostic contribution of the astrocytic biomarker.

The moderate positive correlations observed between GFAP levels and NIHSS scores (ρ = 0.37–0.40) across all time points are consistent with previous studies demonstrating associations between GFAP concentrations and stroke severity [15,16,17]. However, it is noteworthy that some studies have reported no significant correlation between GFAP and NIHSS at admission in moderate-to-severe strokes, while showing stronger associations with radiological measures of infarct size [15]. This discrepancy may reflect the fact that NIHSS primarily captures clinical deficits related to neuronal dysfunction, whereas GFAP specifically reflects astrocytic injury, which may not always correlate linearly with immediate neurological impairment.

The persistence of GFAP-NIHSS correlations at day 6 and discharge suggests that initial GFAP levels may reflect not only the acute injury magnitude but also the trajectory of neurological recovery. Studies examining GFAP dynamics have shown that the rate of change in GFAP levels, rather than absolute concentrations alone, may provide additional prognostic information [18,19]. Specifically, increases in GFAP during the first 24 h post-stroke have been independently associated with larger infarct volumes, higher NIHSS scores, and worse functional outcomes [18]. The stronger correlations observed between GFAP and mRS scores (ρ = 0.48–0.49) compared to NIHSS suggest that GFAP may be particularly valuable for predicting functional disability rather than acute neurological deficits alone. This finding is supported by multiple studies demonstrating that elevated GFAP levels independently predict poor functional outcomes at 3 months, even after adjusting for age, sex, and renal function [15,16,20]. In one large cohort study, patients in the highest GFAP quartile had a 522% increased risk of a poor outcome compared to the lowest quartile [20].

In other studies, when combined with NIHSS, GFAP significantly improved the prediction of poor outcomes (AUC 0.82), suggesting that biomarker-based risk stratification could enhance clinical assessment [20]. Recent evidence indicates that GFAP measured at day 1 post-stroke is an independent predictor of multiple functional outcomes at 3 months, including mRS, Trunk Control Test, Functional Ambulation Classification, and Functional Independence Measure scores [20]. Interestingly, urine GFAP measurements have shown even stronger predictive performance for in-hospital functional outcomes compared to serum GFAP, potentially offering a non-invasive alternative for outcome prediction [21]. However, for longer-term outcomes at 3 months, neurofilament light chain measured at day 7 appears to outperform GFAP as a prognostic biomarker [16].

The observed elevation of serum GFAP in AIS patients (median 132.9 pg/mL) compared to healthy controls (30.0 pg/mL) aligns with the growing body of evidence supporting GFAP as a marker of astrocytic injury following cerebral ischemia [15,16]. The diagnostic accuracy achieved in this study (AUC 0.88) is consistent with previous reports examining GFAP’s ability to differentiate stroke patients from controls, though it should be noted that most prior research has focused on GFAP’s superior performance in distinguishing hemorrhagic from ischemic stroke rather than stroke from healthy individuals [22,23].

The identified cutoff of 71 pg/mL (sensitivity 74% and specificity 92%) provides a balanced threshold for clinical decision making, while the higher cutoff of 150 pg/mL offers exceptional specificity (99%) for rule-in applications. These thresholds are lower than those typically reported for differentiating intracerebral hemorrhage from ischemic stroke, which generally range from 290 to 410 pg/mL, reflecting the distinct pathophysiological mechanisms and magnitude of astrocytic damage between these stroke subtypes [24,25,26]. The lower GFAP concentrations observed in ischemic stroke likely reflect the more gradual release pattern associated with ischemic astrocytic injury compared to the rapid, extensive release occurring with the hemorrhagic disruption of brain tissue [27,28]. The ROC-derived threshold must be seen as exploratory and needs further validation, since the analysis was conducted on the same dataset, introducing the risk of overfitting.

GFAP is an intermediate filament protein that serves as the structural backbone of astrocytes and is released into the bloodstream following astrocytic injury or activation [27]. In ischemic stroke, cerebral ischemia triggers reactive astrogliosis, a complex cellular response involving astrocyte proliferation, hypertrophy, and upregulation of GFAP expression [29,30]. This astrocytic reaction serves dual roles: early reactive astrocytes provide neuroprotective functions by maintaining the blood–brain barrier and limiting lesion expansion, while the later formation of glial scars can impede neural repair [29,31]. The release of GFAP into peripheral blood may occur through multiple mechanisms, including direct cellular damage with membrane disruption, active secretion via extracellular vesicles, and increased blood–brain barrier permeability [27,32]. Recent evidence suggests that GFAP can be transported as cargo within astrocyte-derived extracellular vesicles (ADEVs), providing an additional mechanism for its appearance in peripheral blood [32]. This vesicular transport mechanism may explain why GFAP is detectable even in cases with relatively preserved blood–brain barrier integrity and could account for the dynamic changes in GFAP levels observed during the acute and subacute phases of stroke.

The absence of significant differences in GFAP levels among TOAST subtypes in this study contrasts with the hypothesis that different stroke mechanisms might produce varying degrees of astrocytic injury. The trend toward higher GFAP levels in undetermined etiology strokes (subtype 5) compared to lacunar strokes (subtype 3) and other determined etiologies (subtype 4) is intriguing but requires cautious interpretation, given the small sample sizes in these subgroups.

Limited published data exist regarding GFAP variations across etiological subtypes of ischemic stroke. Most studies have focused on differentiating ischemic from hemorrhagic stroke or examining GFAP in relation to stroke severity and outcomes rather than etiology [22,23,33]. The lack of significant differences observed here may reflect the fact that GFAP release is primarily determined by the extent of tissue injury and astrocytic activation rather than the underlying vascular mechanism. Alternatively, the study may have been underpowered to detect subtle differences between subtypes, particularly for less common categories such as other determined etiology (n = 2).

While this study measured GFAP at a single time point within 24 h of symptom onset, the literature demonstrates that GFAP exhibits a characteristic temporal profile in ischemic stroke. GFAP typically shows an early peak at day 1 post-stroke, distinguishing it from other neuronal injury markers such as NfL, which peaks later at day 7 [16]. This early elevation makes GFAP particularly suitable for acute diagnostic applications and early prognostic assessment.

Studies employing serial GFAP measurements have revealed that the rate of GFAP change provides additional prognostic information beyond single time point measurements [18,19]. The GFAP release rate calculated between admission and immediately after recanalization therapy demonstrated excellent discriminative capacity for clinical outcomes (AUC 0.88), potentially exceeding the predictive value of admission CT imaging scores [19]. Furthermore, monitoring GFAP progression over the first 24 h after intravenous thrombolysis has been shown to independently predict infarct volume, neurological deterioration, and 3-month functional outcomes [18].

The integration of GFAP measurement into clinical stroke protocols holds promise for multiple applications. In the prehospital setting, point-of-care GFAP testing has demonstrated potential for the rapid differentiation of hemorrhagic from ischemic stroke, which could optimize patient triage and enable an earlier initiation of appropriate therapies [10,33,34]. When combined with clinical stroke scales, GFAP testing significantly improves the accuracy of large-vessel occlusion detection, potentially facilitating the direct transfer of appropriate patients to endovascular-capable centers [34].

For ischemic stroke specifically, GFAP could serve as a component of multimodal prognostic models that combine clinical, radiological, and biomarker data to stratify patients for intensive monitoring, aggressive rehabilitation, or enrollment in clinical trials. The complementary nature of GFAP (reflecting astrocytic injury with an early peak) and NfL (reflecting axonal injury with a later peak) suggests that combined biomarker panels may provide more comprehensive prognostic information than either marker alone [15,16].

Several limitations of this study warrant consideration. First, the single-center retrospective design and relatively small sample size, particularly for certain TOAST subtypes, limit the generalizability of findings and statistical power for subgroup analyses. The case-control design comparing patients with acute ischemic stroke to healthy blood donors may lead to an overestimation of diagnostic accuracy. In real-world emergency settings, serum GFAP would be required to discriminate ischemic stroke not only from healthy individuals but also from stroke mimics and other acute neurological conditions, such as seizures, migraines, functional neurological disorders, or metabolic disturbances, which may present with similar clinical symptoms. The absence of such comparator groups in the present study limits the direct generalizability of our diagnostic performance estimates to acute clinical triage scenarios. Future prospective studies conducted in unselected emergency department populations, including patients with suspected stroke and common mimics, are therefore warranted to more accurately define the clinical utility of GFAP as a complementary diagnostic biomarker. Second, GFAP was measured at only one time point within 24 h of admission, precluding assessments of temporal dynamics and rate-of-change metrics that have shown prognostic value in other studies [18,19]. The lack of long-term follow-up data (e.g., 3-month mRS) prevents the evaluation of GFAP’s ability to predict clinically meaningful functional outcomes beyond hospital discharge. Third, the associations observed between serum GFAP concentrations and clinical outcomes, including NIHSS scores and functional status assessed by the modified Rankin scale, should be interpreted cautiously. Important confounding factors known to influence stroke severity and outcome were not fully accounted for in the present analyses [24,33]. Consequently, the observed associations may reflect residual confounding or shared relationships with overall injury severity rather than a direct prognostic effect of GFAP. Future prospective studies incorporating more complete multivariable adjustments, serial GFAP measurements, and longer-term follow-up are required to determine whether GFAP provides independent prognostic information beyond established clinical and imaging markers. Additionally, the exclusion criteria eliminated patients with recent stroke or TIA, potentially limiting applicability to real-world populations which commonly experience recurrent cerebrovascular events. The sample size of the matched cohort was relatively small, particularly after age and sex matching, which limited the statistical power and increased the risk of type II error. In addition, the analyses stratified by TOAST stroke subtype were severely underpowered, with some subgroups comprising only a small number of patients. As a result, these subgroup analyses should be regarded as exploratory and descriptive only. Non-significant trends observed across subtypes cannot be interpreted as meaningful associations, and no subtype-specific conclusions can be drawn from the present data. Larger, adequately powered cohorts are required to reliably assess potential differences in GFAP levels across stroke etiologies. Finally, while the Lumipulse G GFAP assay used in this study is a validated automated platform with excellent analytical performance, different GFAP assays may yield varying absolute concentrations, complicating the direct comparison of cutoff values across studies and limiting immediate clinical implementation without assay-specific validation [16].

## 5. Conclusions

This exploratory study provides evidence that serum GFAP measured within 24 h of symptom onset serves as both a diagnostic marker for acute ischemic stroke and a prognostic indicator of stroke severity and functional disability. The diagnostic accuracy achieved (AUC 0.88) supports GFAP’s potential utility in complementing clinical and radiological assessment, while the results with NIHSS and mRS suggest its value for risk stratification and outcome prediction in selected situations, as discussed. Although GFAP levels did not significantly differ among TOAST etiological subtypes in this cohort, the consistent associations with severity and disability across all stroke types indicate that GFAP primarily reflects the extent of astrocytic injury rather than specific vascular mechanisms.

Future research should focus on prospective multicenter studies with larger sample sizes, serial GFAP measurements to capture temporal dynamics, long-term functional outcome assessments, and the evaluation of GFAP in combination with other biomarkers and clinical variables in multimodal prognostic models. Additionally, studies examining GFAP’s performance in distinguishing ischemic stroke from stroke mimics and other acute neurological conditions are needed to establish its real-world clinical utility. The development and validation of point-of-care GFAP testing platforms could facilitate its integration into emergency and prehospital stroke care pathways, potentially improving patient triage, treatment decisions, and ultimately clinical outcomes.

## Figures and Tables

**Table 1:** Univariate regression analysis. In bold, the statistically significant *p*-values.

Variable	*p*-Value
GFAP	**<0.0001**
Age	0.78
Renal function	0.60
Diabetes mellitus	0.96
Hypertension	0.82
BMI	0.54
Hypercholesterolemia	**0.01**
Atrial fibrillation	0.29
Smoke	0.29
NIHSS at admission	**<0.0001**
MRS at admission	**<0.0001**
TOAST subtype	0.34

**Variable**

***p*-Value**

## Data Availability

Data are available from the corresponding author upon request.
